# Integrated Metagenomic and Metabolomic Analyses Reveal Rhizosphere Soil Microecological Changes in *Thlaspi arvense* L. Lines with Different Alkaloid Contents

**DOI:** 10.3390/microorganisms14030643

**Published:** 2026-03-12

**Authors:** Wenjie Zhang, Chao Fan, Lie Yang, Yan Sun, Lili Tang

**Affiliations:** 1 Heilongjiang Academy of Agricultural Sciences, Harbin 150086, China; 2232234@s.hlju.edu.cn (W.Z.); beyean@163.com (C.F.); yanglie1999@126.com (L.Y.); 2 Academy of Modern Agriculture & Ecology Environment, Heilongjiang University, Harbin 150080, China

**Keywords:** *Thlaspi arvense* L., rhizosphere soil, metagenomics, metabolomics

## Abstract

Pennycress (*Thlaspi arvense* L.), a representative and economically valuable cover crop, supports and enhances key ecological processes throughout its life cycle via its root system. It is hypothesized that pennycress selectively modulates its rhizosphere microbial community through root-derived metabolites, which may influence both the crop’s growth and the subsequent crops in rotation. However, systematic investigations comparing the rhizosphere microbiomes and metabolomes among different pennycress lines remain limited. This study employed metagenomic and metabolomic approaches to examine the dynamic changes in the rhizosphere microbial community and metabolite profiles of three pennycress lines with significantly different total alkaloid contents. The goal was to elucidate the interactions between microbes and metabolites. Results indicated significant differences in microbial community structure across the cultivars. JiL67 maintained stable community diversity, while LiN54 (with the lowest alkaloid content) showed reduced diversity. HeL43 (with the highest alkaloid content) exhibited increased diversity but also potential community homogenization, accompanied by the significant enrichment of microbial taxa capable of alkaloid tolerance. Metabolomic analysis identified metabolites such as Portulacaxanthin II, Oleanolic acid, and Soraphen A as significantly enriched in the rhizosphere soil of pennycress. This study reveals the shifts in rhizosphere microbial communities and metabolites linked to different pennycress lines and uncovers their interactive mechanisms, providing a scientific foundation for developing more economically efficient pennycress cultivation strategies.

## 1. Introduction

Pennycress (*Thlaspi arvense* L.) is a diploid, annual, winter-hardy groundcover plant with abundant germplasm resources. It demonstrates remarkable ecological adaptability and cold tolerance, thriving in various regions and habitats worldwide. As an economic groundcover crop, it holds significant potential for widespread application [1]. Its active root system supports essential ecological processes year-round, offering a practical and effective solution for advancing sustainable agriculture [2,3,4,5]. Pennycress grows well in diverse climates but is most commonly found in temperate regions. Its cold tolerance makes it an ideal candidate for use as a fall-planted cover crop in the U.S. Midwest’s maize–soybean rotation system. Pennycress provides several agronomic and economic benefits: (1) As a cover crop, it improves soil structure while reducing erosion and nutrient loss [6]. (2) Pennycress field residues do not significantly impact the fatty acid content or overall biomass of subsequent soybean crops [7] and effectively suppress weed seed germination and growth [8]. (3) The crop offers an additional revenue stream for farmers. Its seeds, rich in oil (27–39%), are used in soap and lubricant production and serve as a high-quality feedstock for biodiesel production [9]. Additionally, both the whole plant and tender shoots are utilized for food and medicinal purposes. Due to its content of secondary metabolites such as glucosinolates, alkaloids, and coumarins, pennycress is recognized in traditional Chinese medicine for its liver-clearing, vision-improving, digestive-regulating, diuretic, detoxifying, and anti-swelling properties [10]. Despite its substantial economic value, pennycress has long been regarded as a weed in China, hindering its resource development and utilization [11]. Consequently, it is crucial to conduct systematic research on the interactions between pennycress and soil microorganisms, as well as the biosynthesis regulation of its specific metabolites. Such research will provide the theoretical foundation and technical roadmap for its resource utilization.

Plant secondary metabolites are central to chemical defense mechanisms. Among these, allelopathic compounds such as alkaloids, terpenoids, and phenolics play key roles in plant adaptation and defense, enhancing resistance to both biotic and abiotic stresses [12,13]. In plant defense responses, the activation of the jasmonic acid (JA) signaling pathway triggers the coordinated expression of downstream defense genes, driving the synchronous accumulation of secondary metabolites such as terpenoids and alkaloids [14]. The rhizosphere is a key micro-environment where plant–soil interactions occur, serving as a primary site for plant secondary metabolism and energy transformation [15]. Root exudates, such as alkaloids, amino acids, and terpenoids, play a significant role in plant–microbe interactions by attracting and promoting the enrichment of specific microorganisms through mechanisms like structuring, recruitment, and interference. These interactions, in turn, modify the plant’s metabolic profile, enhancing crop yields and the content of bioactive compounds while suppressing pathogenic or harmful microbes [16,17,18]. Rhizosphere microorganisms, as integral components of the microbiome, are pivotal for plant growth and development, biocontrol of pests and diseases, and the accumulation of secondary metabolites [3,19,20]. For example, the core microbial community of the citrus rhizosphere regulates microbe–plant and microbe–microbe interactions, facilitates nutrient acquisition, and promotes plant growth [21]. In sorghum’s early growth stages, stochastic processes significantly influence fungal communities [22]. Rhizosphere microorganisms can also directly inhibit pathogen growth by synthesizing and secreting antimicrobial substances, such as polyketides and terpenoids, serving as an effective disease control mechanism [23,24]. Furthermore, microbial communities can shape host plant metabolism [25]. Thus, understanding the dynamics of the crop rhizosphere microbiome and metabolome is essential for optimizing production practices [26].

From domestic and international germplasm collections, the present study selected one high-alkaloid and two low-alkaloid pennycress accessions with significantly different total alkaloid contents for a three-year continuous cultivation trial, aiming to reveal how line differences in alkaloid production shape rhizosphere microecological processes. We hypothesized that different crop lines shape their rhizosphere microbiome and metabolome in a genotype-dependent manner, with specific microbial and metabolic signatures associated with plant performance. To test this, the specific objectives of this study were: to compare the rhizosphere microbial diversity, community composition, and structure among the three pennycress lines using metagenomic sequencing; to characterize rhizosphere metabolite profiles and identify key metabolites that differ between lines; and to integrate metagenomic and metabolomic data to reveal key interactions and functional pathways within the plant–rhizosphere microbiome system. The results will provide a theoretical foundation for breeding new pennycress varieties and developing tailored cultivation practices, and offer ecological insights that could inform the future development of bioherbicides or microbial inoculants derived from specific metabolites.

## 2. Materials and Methods

### 2.1. Experimental Materials

The three pennycress germplasm accessions selected for this study, designated JiL67, LiN54, and HeL43, were sourced from Jilin, Liaoning, and Heilongjiang provinces in China. A three-year field trial was conducted at the Minzhu Experimental Station of the Heilongjiang Academy of Agricultural Sciences in Harbin (45°49′ N, 126°48′ E; topsoil pH 7.2, organic matter 42 g/kg). Each accession was replicated three times, with an unplanted control plot (CK), resulting in a total of 12 plots (20 m × 20 m = 400 m^2^), spaced 2 m apart. Seeds were sown on 1 September over three consecutive years, with a seeding rate of 1.5 kg/ha and a planting depth of 0.5 cm. All plots were uniformly managed, and plants were harvested around 20 June in the second year after overwintering. Aboveground biomass and rhizosphere soil samples were collected on 10 June 2025 (the third year). All lines were sampled on the same day at the harvest stage (physiological maturity) to ensure phenological consistency, as this stage coincides with peak alkaloid accumulation and represents the optimal time for evaluating both plant yield traits and the potential legacy effects of different genotypes on the soil microecology.

### 2.2. Measurement Indicators and Methods

At pennycress maturity, a 1 m^2^ quadrat was harvested from each plot. The fresh biomass was immediately weighed, and the number of plants within the quadrat was recorded to calculate the fresh weight per plant. Plant height was measured from the cotyledon scar to the apex of the primary branch. After air-drying to a constant weight, dry weight per plant was calculated. For each line, three independent biological replicates (three individual representative plants) were used for biochemical analysis, and no sample pooling was performed. Protein content was quantified using the BCA Protein Assay Kit (Jiangsu Adsion Biotechnology Co., Ltd., Yancheng, China), and total alkaloid content was determined using the Total Alkaloid Content Assay Kit(Jiangsu Adsion Biotechnology Co., Ltd., Yancheng, China), following manufacturers’ instructions.

This study included four treatments: one blank control (CK) and three lines. For each treatment, three biological replicates underwent rhizosphere soil metagenomic sequencing, and six biological replicates underwent rhizosphere soil metabolomic profiling. The corresponding three replicates were used for integrated analysis of metagenomics and metabolomics, while all six replicates were used for independent statistical analysis of the metabolomic data to enhance statistical power. Rhizosphere soil was sampled using the five-point method: the root zone at a 0–10 cm depth was excavated with a trowel. Roots were carefully removed, and the adhering rhizosphere soil was collected by gently shaking the roots (the root-shaking method). The soil samples were sieved through a 20-mesh sieve, placed in centrifuge tubes, flash-frozen in liquid nitrogen, stored at −80 °C, and all statistical analyses of the soil microbial community and metabolite data were performed using biological replicates as independent units.

### 2.3. Metagenome Sequencing and Analysis

DNA was extracted from the soil samples using the E.Z.N.A.^®^ Mag-Bind Soil DNA Kit (Omega Bio-Tek, Norcross, GA, USA), following the manufacturer’s protocol. The concentration and purity of the extracted DNA were measured using a NanoDrop 2000 instrument (Wilmington, DE, USA), and its quality was assessed by 1% agarose gel electrophoresis. DNA samples meeting quality criteria (OD260/280 = 1.8–2.2, OD260/230 ≥ 2.0) were stored at −80 °C for library construction. Sequencing libraries were prepared using the NEB Next^®^ Ultra™ DNA Library Prep Kit for Illumina and sequenced by BMK Cloud Technology (Wuhan, China) Co., Ltd. Qualified DNA was fragmented into ~350 bp segments using a Covaris S2 ultrasonicator (Woburn, MA, USA). The fragmented DNA underwent end repair, A-tailing, adapter ligation, size selection, PCR amplification, and purification to generate the final library. After QC, the libraries were sequenced on an Illumina NovaSeq 6000 platform (San Diego, CA, USA) to generate 150 bp paired-end reads.

### 2.4. Untargeted Metabolomics Assays in the Rhizosphere Soil of the Field Pennycress

For metabolite extraction, 50 mg of soil was weighed into a 2 mL tube. One milliliter of extraction solution (methanol/acetonitrile/water, 2:2:1, *v*/*v*/*v*) containing an internal standard (20 mg/L) was added, and the mixture was vortexed for 30 s. Steel beads were then added, and the sample was homogenized at 45 Hz for 10 min, followed by ultrasonic treatment for 10 min in an ice-water bath. The sample was incubated at −20 °C for 1 h and then centrifuged at 12,000 rpm (4 °C) for 15 min. A 500 μL aliquot of the supernatant was transferred to a microcentrifuge tube and dried in a vacuum concentrator. The dried metabolites were reconstituted in 160 μL of solvent (acetonitrile/water, 1:1, *v*/*v*), vortexed for 30 s, ultrasonicated on ice for 10 min, and centrifuged again (12,000 rpm, 4 °C, 15 min). Finally, 120 μL of the supernatant was transferred to an injection vial. For QC, 10 μL from each sample was pooled to create a QC sample. Metabolomic analysis was performed using an LC-MS/MS system consisting of a Waters Acquity I-Class PLUS UPLC coupled to a Waters Xevo G2-XS QToF mass spectrometer (Milford, MA, USA). Separation was achieved using a Waters Acquity UPLC HSS T3 column (1.8 μm, 2.1 × 100 mm).

### 2.5. Data Analysis

Raw data preprocessing was performed using Microsoft Excel 2019 (Version 2207, Microsoft Corporation, Washington, DC, USA, http://office.microsoft.com/excel (accessed on 10 July 2025)). For the original data of agronomic traits and physiological indices, data collation, outlier screening, missing value imputation, and basic statistical calculations were conducted, accompanied by data format regularization to prepare the datasets for subsequent analyses. For metagenomic and metabolomic data, downstream analyses were implemented using domain-specific bioinformatics pipelines, with the detailed procedures and corresponding software clearly described in this section.

Raw metagenomic sequencing data were quality-filtered using FASTP 0.20.0 to remove low-quality reads and adapter sequences. The obtained clean reads were assembled using MEGAHIT 1.2.9. Open reading frames (ORFs) were predicted using MetaGeneMark 3.38. A non-redundant gene catalog was constructed, and the amino acid sequences were aligned against the Kyoto Encyclopedia of Genes and Genomes (KEGG) (http://www.genome.jp (accessed on 3 October 2025)) database for functional annotation, and against the NCBI non-redundant protein (NR) (https://www.ncbi.nlm.nih.gov (accessed on 5 October 2025)) database for taxonomic assignment. Statistical analyses were conducted using IBM SPSS Statistics (Version 23), including one-way ANOVA and Duncan’s multiple comparison tests (*p* < 0.05). Data were further analyzed and visualized with GraphPad Prism 2023 to illustrate changes in key indicators. Alpha diversity indices (Shannon, Chao1, and Pielou’s evenness) were calculated using R 4.3.2. Principal coordinate analysis (PCoA) based on Bray–Curtis distances and redundancy analysis (RDA) were performed to visualize sample dissimilarities. PERMANOVA was performed using the vegan R package (version 2.6-4) to test for significant differences in community structure among treatments. Mantel test, Metastats analysis, and LEfSe (LDA score > 2.0) were conducted to identify differentially abundant features and biomarkers. All statistical analyses were performed with biological replicates as independent units. The metagenomic results were visualized using Origin 2022 and BioCloud (https://www.biocloud.net/ (accessed on 25 October 2025)). For both microbial community and metabolomic data, multiple comparison corrections were applied to control the false discovery rate (FDR).

Metabolomic profiling was performed using LC-MS/MS. Raw data, acquired with MassLynx V4.2, were processed using Progenesis QI software V3.0 for peak picking, alignment, and other data processing operations. Metabolite identification was performed based on the online METLIN database, public databases, and a custom-built library within Progenesis QI, accompanied by theoretical fragmentation pattern recognition. After normalizing peak areas to the total ion count, subsequent analyses were conducted. Principal component analysis (PCA) and Spearman correlation analysis were used to evaluate intra-group repeatability and quality control (QC) sample stability. Compound identities were cross-referenced against the KEGG, Human Metabolome Database (HMDB) (https://hmdb.ca/ (accessed on 27 October 2025)), and LIPID MAPS Structure Database (Lipidmaps, Cardiff, UK) (https://lipidmaps.org/ (accessed on 29 October 2025)) for classification and pathway information. Based on group assignments, fold changes (FC) were calculated and compared, and Student’s *t*-test was applied to determine significance (*p*-value) for each compound. Orthogonal partial least squares-discriminant analysis (OPLS-DA) was performed using the ropls R package(version 1.32.0), with model validity confirmed by 200 permutation tests. Variable importance in projection (VIP) scores were derived from multiple cross-validation. Differential metabolites were identified using thresholds of FC > 1, *p*-value < 0.05, and VIP > 1. Enrichment of these metabolites in KEGG pathways was assessed using a hypergeometric test.

## 3. Results

### 3.1. Comparison of Rhizosphere Microecology Between High- and Low-Alkaloid Pennycress Accessions

#### 3.1.1. Agronomic Traits and Physiological Indicators

The agronomic traits and physiological indicators of the three pennycress lines are summarized in Table 1. The three pennycress accessions used in this study were designated as H (high-alkaloid line, HeL43), L1 (low-alkaloid line, JiL67), and L2 (low-alkaloid line, LiN54) according to their total alkaloid contents. Among them, H’s total alkaloid content (2.65 mg/g) was significantly higher (*p* < 0.05) than that of L1 (1.50 mg/g) and L2 (1.40 mg/g). In contrast, H exhibited the highest mean values for the dry-to-fresh weight ratio (D/F) and protein content. However, no significant differences were observed among the lines in terms of plant height, D/F, or protein content.

#### 3.1.2. Variation in the Rhizosphere Microbial Community

**Figure 1:**
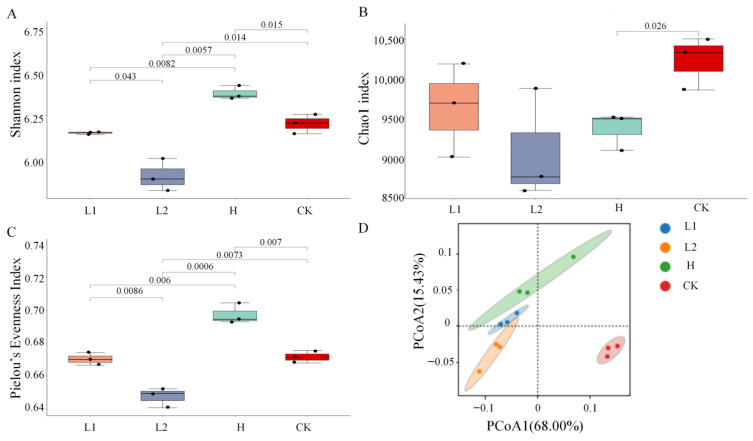
Microbial community diversity in pennycress samples. (**A**) Shannon index. (**B**) Chao1 index. (**C**) Pielou’s Evenness Index. (**D**) Microbial community clustering diagram based on Bray–Curtis PCoA. CK: unplanted control plot.

After QC, Illumina high-throughput sequencing generated a total of 79,472,134,434 clean reads. For all samples, Q30 scores exceeded 92% (Supplementary Table S1), confirming the high accuracy of the sequencing data. Rarefaction curves for each sample plateaued, indicating that the sequencing depth was sufficient to capture the microbial diversity present (Supplementary Figure S1). Alpha diversity analysis of the soil microbial community is presented in Figure 1. Compared with the two low-alkaloid lines and CK (the unplanted control), After H cultivation, both the Shannon index (*p* < 0.05) and Pielou’s evenness index (*p* < 0.01) increased significantly, while the Chao 1 index declined (*p* < 0.05). This suggests that the observed increase in community diversity was largely driven by enhanced evenness rather than richness. These changes indicate a homogenization process within the community, characterized by a more uniform species distribution, likely due to selection against rare species.

Principal coordinate analysis (PCoA) revealed clear separation among the four groups, with PCoA1 and PCoA2 explaining 68.00% and 15.43% of the variance, respectively (Figure 1D). This indicates that inter-group differences were the primary driver of microbial community variation, with H exhibiting the greatest separation from L1, L2, and CK. This pattern was strongly supported by ANOSIM based on Bray–Curtis distances (R = 0.809, *p* = 0.001; Supplementary Figure S2), confirming significant structural differences between groups. These results demonstrate that pennycress cultivation significantly altered the composition of the soil microbial community.

#### 3.1.3. Composition of the Rhizosphere Microbial Community

**Figure 2:**
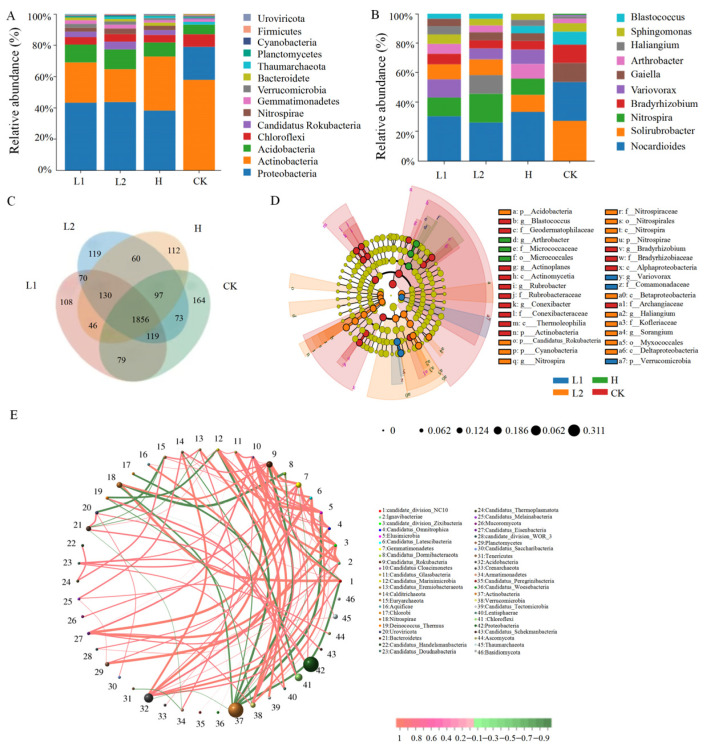
Composition of microbial community in pennycress samples. The composition of the rhizosphere soil microbial community at the phylum (**A**) and genus (**B**) levels in pennycress samples (only the top 10 are shown). (**C**) Venn diagram depicting the overlap of genera across different groups. (**D**) Cladograms from LEfSe analysis showing microbial taxa with differential abundance across groups at various taxonomic levels. (**E**) Correlation network analysis of microbial taxa in different groups.

At the phylum level of microbial communities, bacteria dominate the rhizosphere soil (Supplementary Figure S3). In the control soil (CK), Actinobacteria, Proteobacteria, and Chloroflexi were the dominant phyla. In contrast, the dominant phyla in the pennycress-planted soils (L1, L2, and H) were Proteobacteria, Actinobacteria, and Acidobacteria (Figure 2A). To facilitate an in-depth analysis of bacterial community variation across the pennycress lines, the communities were profiled at the genus level. H exhibited the lowest number of genera, as shown in Figure 2C (A total of 2516, 2524, 2464, and 2551 genera were detected in the L1, L2, H, and CK groups, respectively). Across the four groups, the top ten genera in terms of abundance were largely consistent, though their relative abundances varied (Figure 2B). In CK, *Solirubrobacter*, *Nocardioides*, *Gaiella*, *Bradyrhizobium*, and *Blastococcus* exhibited relatively higher abundances. In H, *Nocardioides*, *Solirubrobacter*, *Nitrospira*, *Arthrobacter*, and *Variovorax* were the most abundant genera. LEfSe analysis revealed significant differences in the relative abundance of microbial communities between each of the three pennycress-planted groups and CK (Figure 2D,E). LEfSe identified a total of 34 biomarkers (LDA score > 4.0) across the four sample groups (Supplementary Figure S4). Specifically, the CK community comprised 14 taxonomic units, with Actinobacteria being the primary contributor. Compared with the low-alkaloid lines, the community of H contained 3 units, primarily contributed by the order Micrococcales. In the primary enriched communities of the rhizosphere, a positive correlation was observed between Nitrospirae and beneficial bacteria such as Acidobacteria and Candidatus Rokubacteria, while Actinobacteria showed a negative correlation with Nitrospirae.

#### 3.1.4. Potential Functional Pathways of the Rhizosphere Microbiome

**Figure 3:**
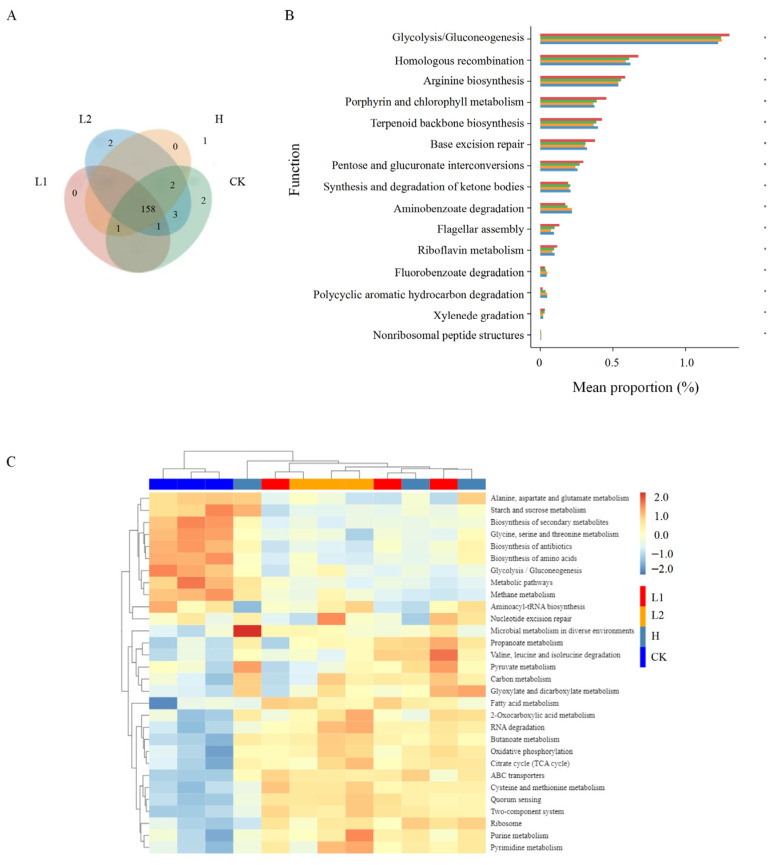
Analysis of potential functional pathways in soil microorganisms. (**A**) Venn diagram of KEGG level 3 pathways across all samples. (**B**) Bar chart showing the top 15 significantly different functions based on the Wilcoxon rank-sum test. For each function, the left bar indicates mean relative abundance (*x*-axis) for each group (blue: L1; orange: L2; green: H; red: CK)), the center shows significance level (*, *p* < 0.05 ), and the right number represents the exact *p*-value. Functions are ranked first by *p*-value (descending) and then by abundance (highest to lowest). (**C**) Heatmap of the top 30 differentially abundant functional pathways.

As shown in Figure 3A, the four soil sample groups were assigned to a total of 169 KEGG level 3 pathways, with 160, 166, 161, and 167 pathways detected in L1, L2, H, and CK, respectively. Notably, LiN54 harbored two unique pathways: Glycosphingolipid biosynthesis–ganglio series and Glycosphingolipid biosynthesis–globo and isoglobo series. Conversely, CK contained two distinct pathways: Mitophagy—yeast and SNARE interactions in vesicular transport. Based on annotation results (Figure 3B), significant differences in potential functions were observed across the four groups, particularly in: Glycolysis/Gluconeogenesis, Homologous recombination, Arginine biosynthesis, Porphyrin and chlorophyll metabolism, and Terpenoid backbone biosynthesis. Additionally, the relative abundances of genes involved in ketone body synthesis and degradation, aminobenzoate degradation, fluorobenzoate degradation, and polycyclic aromatic hydrocarbon degradation were significantly higher after pennycress cultivation. A heatmap of the top 30 differentially abundant functional pathways is shown in Figure 3C. The results revealed that after pennycress cultivation, the L1 group was enriched in Propanoate metabolism, Valine, leucine, and isoleucine degradation, and Fatty acid metabolism. The L2 group exhibited enrichment in 2-Oxocarboxylic acid metabolism, RNA degradation, and Pyrimidine metabolism. In the H group, enrichment was observed in Microbial metabolism in diverse environments, Pyruvate metabolism, and Glyoxylate and dicarboxylate metabolism.

#### 3.1.5. Non-Targeted Soil Metabolite Analysis

Correlation analysis between the three pennycress lines and the control soil samples revealed correlation coefficients greater than 0.88, indicating a high similarity in metabolite expression levels across the samples (Supplementary Figure S5). According to the HMDB (covering the top 20 categories by metabolite count), the primary metabolites in pennycress rhizosphere soil included fatty acyls, organooxygen compounds, carboxylic acids and derivatives, prenol lipids, steroids, and steroid derivatives (Supplementary Figure S6).

**Figure 4:**
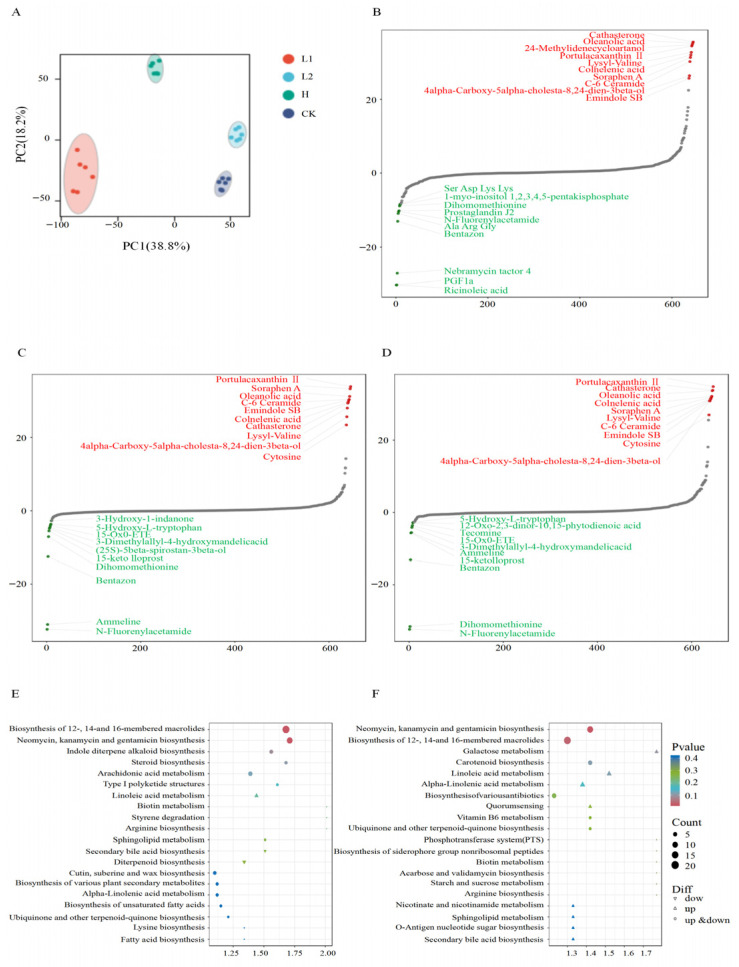
(**A**) PCA of metabolites across four groups. (**B**) Dynamic distribution map of metabolite content differences between CK and L1. (**C**) Dynamic distribution map of metabolite content differences between CK and L2. (**D**) Dynamic distribution map of metabolite content differences between CK and H. The horizontal axis represents the cumulative number of substances sorted by fold change in ascending order, while the vertical axis displays the logarithm of fold change to base 2. (**E**) Metabolic pathway enrichment analysis diagram for L1 versus H. (**F**) Metabolic pathway enrichment analysis for L2 versus H. Each point in the diagram represents a KEGG pathway, with the *x*-axis denoting the enrichment factor (Rich factor) and the *y*-axis showing the pathway name.

PCA showed significant differences in rhizosphere soil metabolite composition among L1, L2, CK, and H groups (Figure 4A). The first two principal components, PC1 and PC2, explained 38.8% and 18.2% of the total variance, respectively. Differential metabolite analysis between H and the other three groups revealed distinct metabolic profiles. A total of 343, 369, and 366 differentially expressed metabolites (DEMs) were identified in comparisons with L1, L2, and CK, respectively (Supplementary Table S2). Notably, the number of upregulated DEMs was significantly greater than the number of downregulated DEMs in all three lines. Venn diagram analysis (Supplementary Figure S7) indicated that 216 differential metabolites were common to all three pennycress lines when compared to the CK. This suggests that the differences among pennycress samples were primarily due to metabolite abundance rather than compositional profile.

FC values for metabolites were calculated for each comparison group, and metabolites were then sorted in ascending order of FC to generate a dynamic distribution plot of metabolite differences. Compared with CK, L1, and L2, metabolites such as Portulacaxanthin II, Cathasterone, Oleanolic acid, Colnelenic acid, and Soraphen A were significantly upregulated, while metabolites like N-Fluorenylacetamide, Dihomomethionine, Bentazon, 15-keto lloprost, and Ammeline were significantly downregulated in H (Figure 4B–D). Among these differential metabolites, the three pennycress lines shared compounds enriched with Portulacaxanthin II (a carotenoid with anti-inflammatory and antioxidant activities), Oleanolic acid (an important plant secondary metabolite), and Soraphen A (a polyketide macrolide antibiotic produced by Myxobacteria). These identifications are tentative annotations based on database matching and MS/MS spectral evidence, as described in the Methods section.

To elucidate specific metabolic changes in the pennycress rhizosphere, pathway enrichment analysis was performed using the KEGG database on the differential metabolite sets from each of the three lines. The pathways “Neomycin, kanamycin, and gentamicin biosynthesis” and “Biosynthesis of 12-, 14-, and membered macrolides” were consistently and significantly enriched in H among all groups (Figure 4E,F).

### 3.2. Comparison of Differences in Two Low-Alkaline Containing Lines

There were no significant differences in plant height, dry-to-fresh weight ratio, protein content, or total alkaloid content between the two low-alkaloid lines (Table 1). However, the cellulose content of L2 (2.77 mg/g) was significantly higher than that of L1 (1.63 mg/g).

Analysis of the soil microbial community revealed that in L1, neither the Shannon nor the Chao 1 index showed significant changes (*p* > 0.05), indicating stable overall community diversity (Figure 1). In contrast, cultivation of L2 resulted in significant reductions in both the Shannon index (*p* < 0.05) and Pielou’s evenness index (*p* < 0.01), reflecting decreased diversity, while the Chao 1 index remained unchanged, suggesting no shift in species richness.

LEfSe analysis of the rhizosphere microbial community in L1 and L2 revealed that, among the biomarkers with an LDA threshold > 4.0, the L1 community contained 3 units, predominantly from the family Comamonadaceae (Figure 2). The L2 community included 14 units, with major contributions from the class Betaproteobacteria, genus *Nitrospira*, order Myxococcales, class Deltaproteobacteria, and family Nitrospiraceae.

Dynamic analysis of L1 and L2 metabolites revealed significant differences in their levels compared to CK. In L1, several metabolites—including Cathasterone, Oleanolic acid, 24-Methylidenecycloartanol, Portulacaxanthin II, and Lysyl-Valine—were significantly up-regulated, while others—such as Ricinoleic acid, PGF 1a, Nebramycin factor 4, Bentazon, and Ala Arg Gly—were significantly downregulated (Figure 4B). In L2, metabolites such as Portulacaxanthin II, Soraphen A, Oleanolic acid, C-6 Ceramide, and Emindole SB were significantly upregulated, while N-Fluorenylacetamide, Ammeline, Bentazon, Dihomomethionine, and 15-keto lloprost were significantly downregulated (Figure 4C).

### 3.3. Joint Analysis of Soil Microorganisms and Metabolites

**Figure 5:**
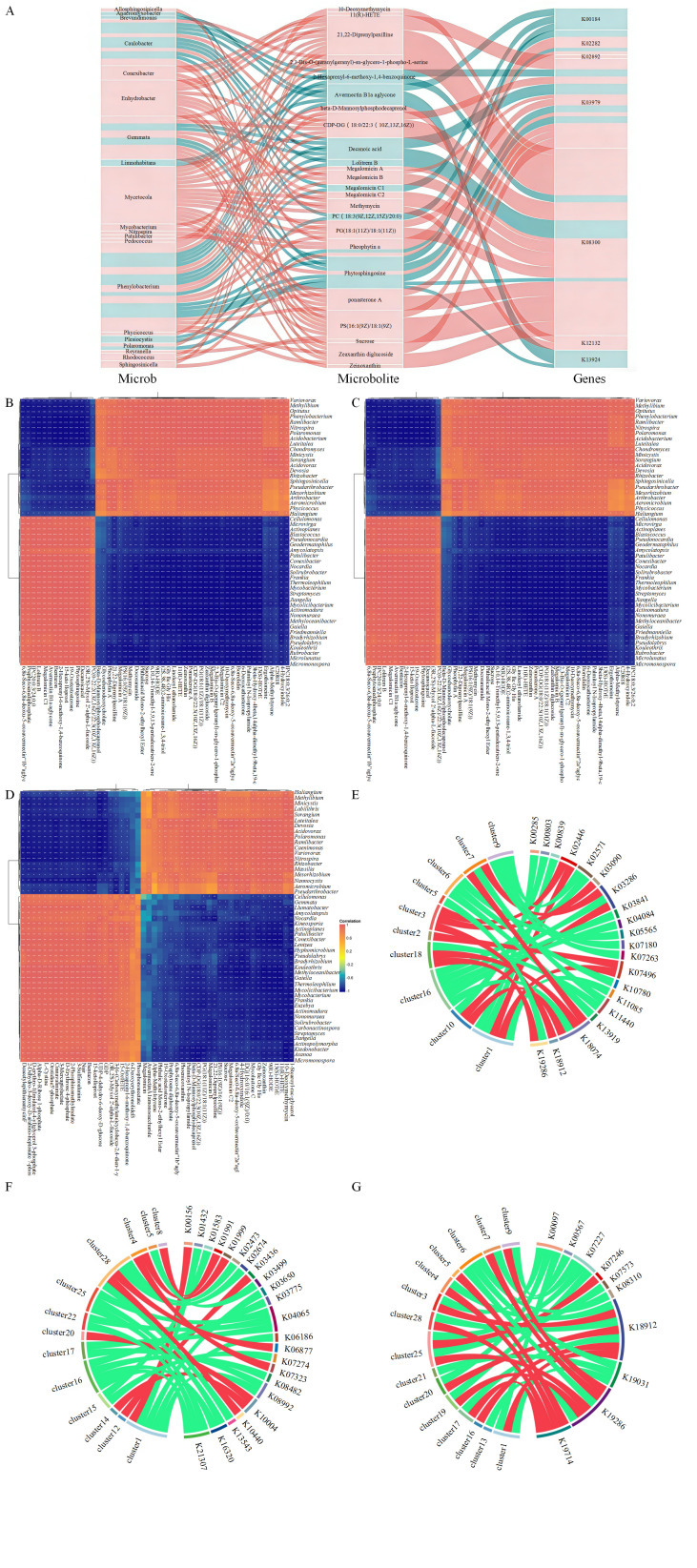
Integrated analysis of soil microorganisms and metabolites. (**A**) Sankey diagram of species-functional gene-metabolite associations. Red denotes positive correlation, green denotes negative correlation. (**B**) Heatmap correlating the abundance of differentially abundant metabolites and microorganisms in L1. (**C**) Heatmap correlating the abundance of differentially abundant metabolites and microorganisms in L2. (**D**) Heatmap correlating the abundance of differentially abundant metabolites and microorganisms in H. Rows represent differentially abundant microbial species; columns represent differentially abundant metabolites. *, *p* < 0.05; **, *p* ≤ 0.01; ***, *p* ≤ 0.001. (**E**) String diagram of metabolite-microbial species-functional gene correlations for L1. (**F**) String diagram of metabolite-microbial species-functional gene correlations for L2. (**G**) String diagram of metabolite-microbial species-functional gene correlations for H. The left half of the string diagram represents metabolites, while the right half represents microbial species. A wider string width indicates a higher frequency of association with that metabolite or microorganism. Red: positive correlation; green: negative correlation.

To explore the associations among differential metabolites, microorganisms, and functional genes, a Sankey diagram was constructed for microbial species–functional gene–metabolite correlation analysis (|r| > 0.8, *p* < 0.05; Figure 5A). Overall, the correlation networks exhibited more positive than negative links. The microbial species Phenylobacterium, Caulobacter, Mycetocola, and Gemmate displayed the highest number of significant correlations with differential metabolites, while metabolite 21,22-Diprenylpaxilline and PS (16:1(9Z)/18:1(9Z)) were most closely linked to a variety of microbial species. Notably, nearly all significant differential metabolites were associated with the functional gene K08300 (ribonuclease E [EC:3.1.26.12]) in the RNA degradation pathway.

Pearson correlation analysis was performed to assess the relationship between the abundances of differential metabolites and microbial taxa, with corresponding heatmaps generated. Among the 50 bacterial genera most strongly correlated with differential metabolites, each genus in L1 was significantly correlated with metabolites such as Sucrose, 11(R)-HETE, 15-keto lloprost, Bentazon, and 13(S)-HOTrE (Figure 5B). In L2, genera were most significantly correlated with metabolites 11(R)-HETE, 15-keto lloprost, Bentazon, 15-Oxo-ETE, and Glycochenodeoxycholate (Figure 5C), while in H, the genera showed significant correlation with metabolites 9(R)-HODE, 11(R)-HETE, 15-keto lloprost, Bentazon, and 13(S)-HOTrE (Figure 5D). The metabolites 11(R)-HETE, 15-keto lloprost, and Bentazon appeared to be closely associated with microbial communities during pennycress cultivation.

A joint analysis of metabolites and functional genes was conducted using hierarchical clustering and correlation analysis. A chord diagram was constructed to visualize the associations between the top 30 differential metabolites and functional genes. In L1, the greatest number of metabolites showed significant correlations with K18074 (terephthalate 1,2-dioxygenase oxygenase component alpha subunit [EC:1.14.12.15]), and most of these correlations were positive (Figure 5E). In L2, most metabolites were significantly correlated with K04065 (hyperosmotically inducible periplasmic protein) and K21307 (sulfite dehydrogenase (quinone) subunit SoeA [EC:1.8.5.6])—both from the phylum Actinobacteria—and these correlations were predominantly negative (Figure 5F). In H, the largest number of metabolites correlated significantly with K18912 (gamma-glutamyl hercynylcysteine S-oxide synthase [EC:1.14.99.50]), with a predominance of positive correlations (Figure 5G).

## 4. Discussion

The rhizosphere, influenced by root exudates, exhibits significant differences in microbial community abundance and diversity compared to the bulk soil matrix [27]. Among these, plant allelopathic effects significantly alter the structure and biological activity of rhizosphere microbial communities [28,29]. Pennycress, known for its strong allelopathic effects [30], significantly altered the soil microbial communities upon cultivation. Compared to the bulk soil control (CK), all three pennycress lines exhibited notable differences in their associated microbial communities. The alpha diversity of the rhizosphere community in L1 remained stable, whereas it decreased in L2. Conversely, H exhibited increased diversity along with signs of community homogenization. These findings suggest that the pennycress line is the primary factor driving the distinct rhizosphere microbiome structures observed among the groups [31,32].

The rhizosphere microbial community in this study was dominated by Proteobacteria, Actinobacteria, and Acidobacteria, which aligns with previous research [33,34]. Following pennycress cultivation, the relative abundances of Acidobacteria and Nitrospirae, along with the dominant Proteobacteria, were significantly higher than those in the bulk soil control (CK). These phyla are recognized for their adaptability and role in decomposing complex organic matter, contributing significantly to soil carbon balance [35,36,37]. Their enrichment may suggest enhanced environmental adaptability in pennycress-treated soil.

Predictive analysis of metagenomic data revealed that, after pennycress cultivation, the rhizosphere microbial community exhibited significantly higher relative abundances of genes related to ketone body synthesis and degradation, aminobenzoate degradation, fluorobenzoate degradation, and polycyclic aromatic hydrocarbon degradation. This suggests an enhanced capacity for fat metabolism and the breakdown of aromatic compounds. The enrichment of pathways such as propanoate metabolism, valine, leucine, and isoleucine degradation, and fatty acid metabolism in L1 suggests that its rhizosphere is involved in energy metabolism and the decomposition and utilization of complex organic carbon sources. Additionally, the enrichment of 2-oxocarboxylic acid metabolism, RNA degradation, and pyrimidine metabolism in L2 indicates that its rhizosphere is engaged in central metabolism and genetic material turnover, essential for maintaining fundamental cellular processes and dynamic equilibrium. In H, the order Micrococcales was a primary contributor, known for its roles in nitrogen cycling [38], organic matter degradation, glucose utilization [39], and bioremediation [40]. Furthermore, the rhizosphere of H also harbored a higher abundance of bacteria associated with alkaloid degradation, such as *Arthrobacter*, *Variovorax*, and *Nocardioides* [36,41], along with the predicted enrichment of pathways related to microbial metabolism in diverse environments, pyruvate metabolism, and glyoxylate and dicarboxylate metabolism. This combined microbial profile may be attributed to the higher alkaloid content in H compared to the other lines. Moreover, in the pennycress rhizosphere, Nitrospirae was positively correlated with putative beneficial taxa such as Acidobacteria and Candidatus Rokubacteria. This correlation suggests that the microbial community may have gained functional efficiency and stability following pennycress cultivation.

Metabolite analysis revealed differences in rhizosphere metabolites among the pennycress lines. PCA indicated that pennycress cultivation significantly altered the soil metabolic composition, further distinguishing the metabolic profiles of the different lines. Compared to CK, the rhizospheres of the three pennycress-planted groups were enriched in specific metabolites: Portulacaxanthin II (a terpenoid known for enhancing plant stress resistance and antioxidant capacity [24,42]), Oleanolic acid (a pentacyclic triterpenoid with anti-inflammatory and antibacterial properties [43,44]), and Soraphen A (a polyketide macrolide antibiotic produced by myxobacteria, exhibiting potent antibacterial activity [45]). Among these, 24-methylidenecycloartanol (a terpenoid linked to plant stress resistance [46]), which was abundant in the L1 line, and the significantly downregulated nebramycin factor 4 (an aminoglycoside antibiotic [47]), collectively may indicate a shift in the rhizosphere from intense microbial antagonism to an adaptive mode dominated by plant structural reinforcement. In the L2 line, the rhizosphere was enriched with cytosine (a nitrogenous heterocyclic compound derived from nucleic acid degradation [48]), while the exogenous pollutant Ammeline [49] was significantly downregulated. This pattern indicates that the soil is transitioning from a disturbed to a more bioactive and healthy state. In the H line, the increased abundance of cytosine, coupled with the downregulation of Dihomomethionine (a sulfur-containing amino acid), suggests active metabolic and stress-resistant processes [50].

Association analysis between microbial taxa, functional genes, and metabolites revealed strong links between differential soil metabolites and microbial communities. The concurrent enrichment of Phenylobacterium (an efficient degrader of aromatic compounds [51]) and the fungal metabolite 21,22-diprenylpaxilline (an indole diterpenoid alkaloid) indicates a statistical association between bacterial and fungal populations in the pennycress rhizosphere. KEGG enrichment analysis of these metabolites identified the biosynthesis of type II polyketide products (a fungal-associated pathway for broad-spectrum antibiotics [52,53]) as the most significantly altered. Together with the co-enrichment of *Mycetocola* (known for its close fungal associations), this suggests a possible correlation with enhanced soil microbiome antibacterial potential following pennycress cultivation.

Additionally, *Caulobacter* (specializing in attachment under oligotrophic conditions [54]) and *Gemmate* (a fast-growing bacterium potentially symbiotic with *Nocardioides* [55]) were significantly associated with the high abundance of PS (16:1(9Z)/18:1(9Z)), a marker lipid for apoptosis, and ribonuclease E in the rhizosphere soil [56]. This suggests that the pennycress rhizosphere may establish an efficient organic nutrient recycling system driven by specific microbes, underpinned by high rates of cellular turnover. Both 21,22-diprenylpaxilline and PS (16:1(9Z)/18:1(9Z)) were positively correlated with all differentially abundant microbial taxa. Since many of these genera promote plant growth, this suggests that higher levels of these metabolites may enhance the proliferation of beneficial genera in the rhizosphere soil. In H, which exhibited higher total alkaloid content, the concentrations of Portulacaxanthin II and Oleanolic acid in the rhizosphere soil were significantly elevated. This suggests that increased alkaloid synthesis in this group may alter the composition of root exudates, exerting selective pressure on the rhizosphere microbial community and specifically enriching microorganisms with alkaloid tolerance/degradation abilities and specific secondary metabolic potential (e.g., *Arthrobacter*, *Variovorax*, *Nocardioides*). Concurrently, the upregulation of genes related to the synthesis and degradation of ketone bodies and the biosynthesis of type II polyketide products suggests that microbes redirected carbon flux, leading to the enrichment of Soraphen A—an antibiotic polyketide macrolide with both antibacterial and herbicidal properties—in the rhizosphere.

While this study has hypothesized connections between some metabolites and microorganisms, the interactions among microbes remain inadequately explored. Future research could be strengthened by integrating enzymatic activity assays in the pennycress rhizosphere and adopting a tripartite analytical framework linking microbes, enzymes, and metabolites. This approach would provide more comprehensive insights into enhancing soil quality and optimizing pennycress agronomic practices.

### Limitations

Several limitations of this study should be acknowledged. First, this study was conducted in Harbin at the pennycress maturity stage, which may limit the applicability of the results across different environments and growth stages. Second, the sample size was relatively small (n = 3), which restricts the statistical power and scope of inference. Third, this was an observational study based on correlations, and thus causal relationships between rhizosphere metabolites, microbial communities, and pennycress traits could not be firmly established. Future studies involving different regions, various growth stages of pennycress, increased sample sizes, and manipulative experiments are required to confirm the reliability of these findings and further clarify the underlying causal mechanisms.

## 5. Conclusions

This study integrated metagenomic and rhizosphere metabolomic analyses to reveal the changes in the rhizosphere soil microecology of pennycress varieties with different total alkaloid contents. Compared to the two lines with lower total alkaloid content, H, which exhibits a higher total alkaloid content, likely exerts strong selection pressure in its rhizosphere, thereby enriching specific microbial taxa with alkaloid tolerance. These enriched microbes may adapt to this chemical stress by reshaping their metabolomes and activating specific secondary metabolic pathways, such as antibiotic biosynthesis pathways, thus gaining a competitive advantage in the rhizosphere. In summary, inter-varietal differences in secondary metabolites, particularly alkaloids, may represent a key driver of the lineage-specific assembly of the pennycress rhizosphere microbiome. Our findings provide novel insights into the metabolite-mediated interactions between pennycress lines and their associated rhizosphere communities.

## Figures and Tables

**Table 1:** Agronomic traits and physiological indicators.

Lines	L1	L2	H
Plant Height (cm)	64.00 ± 9.64 a	74.67 ± 20.01 a	67.33 ± 3.06 a
Fresh weight (g)	53.00 ± 25.08 a	40.35 ± 13.54 a	41.10 ± 18.86 a
Dry weight (g)	15.25 ± 4.89 a	13.55 ± 2.96 a	15.17 ± 7.17 a
D/F Ratio (%)	0.30 ± 0.05 a	0.35 ± 0.05 a	0.37 ± 0.05 a
Protein content (mg/g)	150.68 ± 19.73 a	161.15 ± 5.10 a	173.42 ± 1.44 a
Total alkaloids (mg/g)	1.50 ± 0.40 b	1.40 ± 0.49 b	2.65 ± 0.06 a
Cellulose content (mg/g)	1.63 ± 0.20 b	2.77 ± 0.13 a	1.77 ± 0.43 ab

Different letters following numerical values indicate significant differences (*p* < 0.05), while identical letters denote no significant differences (*p* > 0.05).

## Data Availability

Raw readings of metagenomic and metabolomic data were submitted to the Sequence Read Archive (SRA) for the NCBI database (Accession Number: PRJCA041574 and PRJCA051630).

## Supplementary Materials

The following supporting information can be downloaded at: https://www.mdpi.com/article/10.3390/microorganisms14030643/s1. Table S1: Field pennycress rhizosphere soil microbial metagenome sequencing data statistics; Table S2. Difference significance analysis results of metabolite; Figure S1: Dilution curve of sample microbial communities; Figure S2: Species inter-sample distance boxplot. The vertical axis represents the Beta distance. The box plot above “Between” indicates the Beta distance data for all inter-group samples, while the subsequent box plots show the Beta distance data for intra-group samples within different groupings. In the Anosim analysis, an R-value closer to 1 suggests that inter-group differences are greater than intra-group differences, whereas a smaller R-value indicates no significant distinction between inter-group and intra-group differences. A *p*-value less than 0.05 indicates high reliability of the test; Figure S3: I Kingdom-level composition of the rhizosphere soil microbial community; Figure S4: Cladograms from LEfSe analysis showing microbial taxa with differential abundance across groups at various taxonomic levels; Figure S5: Inter-sample Correlation Heatmap; Figure S6: Primary metabolites in the rhizosphere soil of pennycress; Figure S7: Venn diagram of differentially expressed metabolites.
