# Supplementary material for: Integrated Metagenomic and Metabolomic Analyses Reveal Rhizosphere Soil Microecological Changes in Thlaspi arvense L. Lines with Different Alkaloid Contents
Source: Microorganisms. 2026 Mar 12;14(3):643. doi: 10.3390/microorganisms14030643 (PMC13028767; doi:10.3390/microorganisms14030643)
Supplement: Supplementary file 1 [file microorganisms-14-00643-s001.zip › microorganisms-4166102-supplementary figure.pdf]

microorganisms

Integrated Metagenomic and Metabolomic Analyses Reveal Rhizosphere Soil Microecological Changes in *Thlaspi arvense* L. Lines with Different Alkaloid Contents

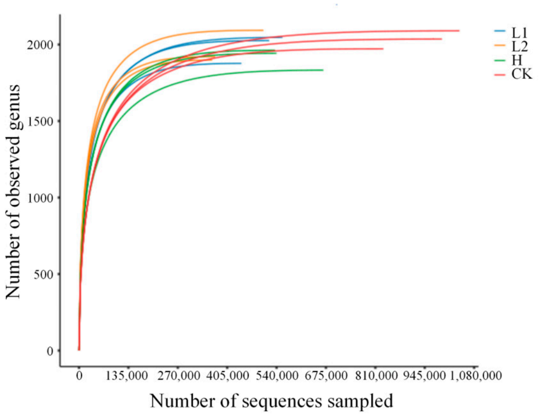

Figure S1. Dilution curve of sample microbial communities

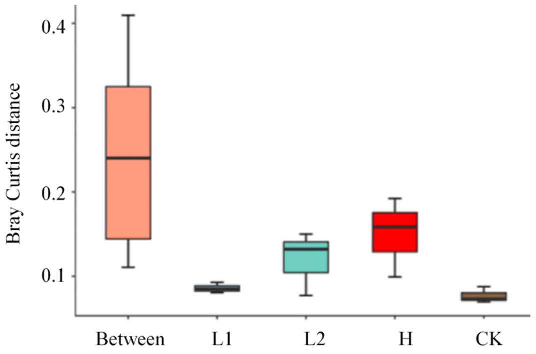

Figure S2. Species inter-sample distance boxplot. The vertical axis represents the Beta distance. The box plot above "Between" indicates the Beta distance data for all inter-group samples, while the subsequent box plots show the Beta distance data for intra-group samples within different groupings. In the Anosim analysis, an R-value closer to 1 suggests that inter-group differences are greater than intra-group differences, whereas a smaller R-value indicates no significant distinction between inter-group and intra-group differences. A P-value less than 0.05 indicates high reliability of the test.

 https://doi.org/10.3390/microorganisms14030643

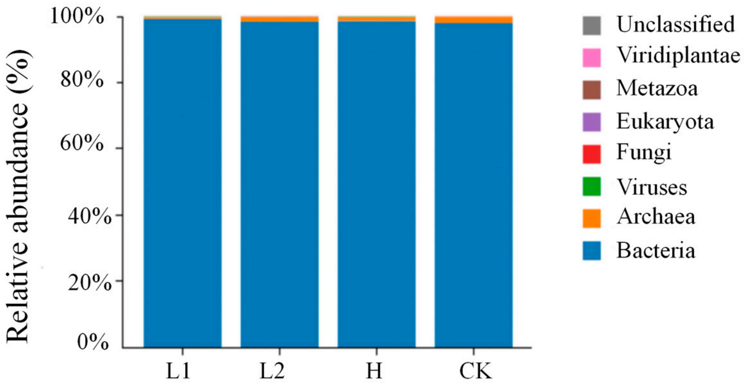

Figure S3. Kingdom-level composition of the rhizosphere soil microbial community.

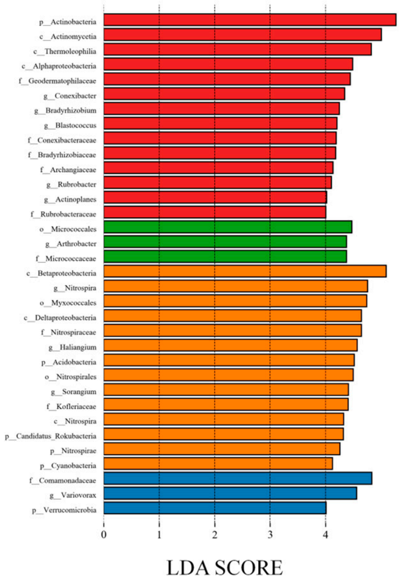

Figure S4. Cladograms from LEfSe analysis showing microbial taxa with differential abundance across groups at various taxonomic levels.

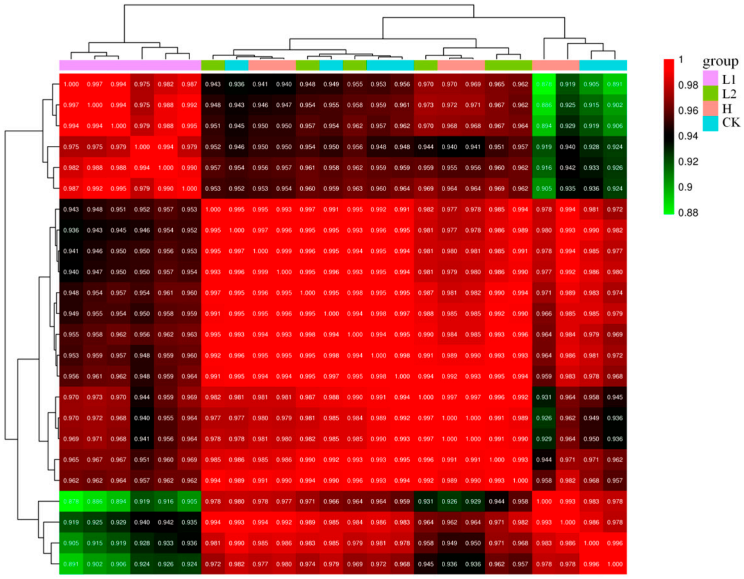

Figure S5. Inter-sample Correlation Heatmap.

https://doi.org/10.3390/microorganisms14030643

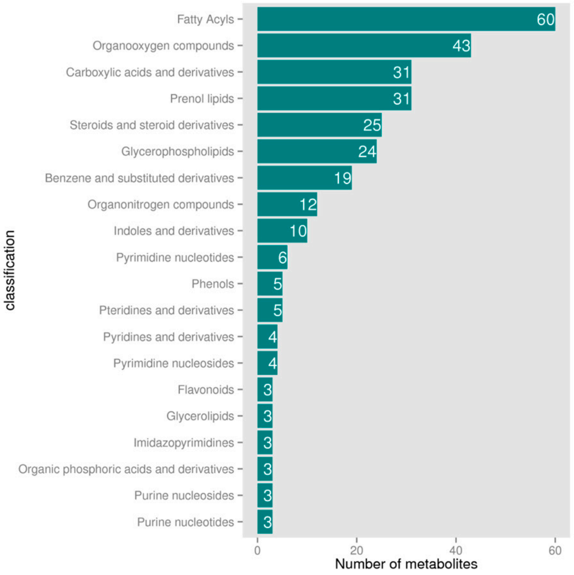

Figure S6. Primary metabolites in the rhizosphere soil of pennycress.

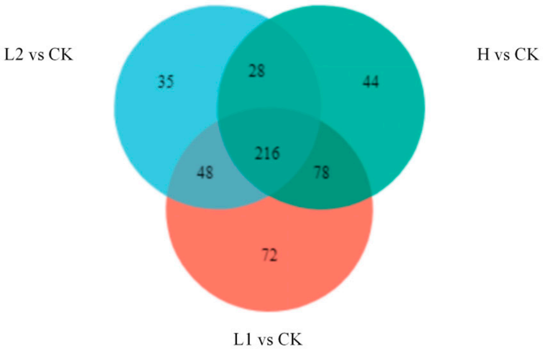

Figure S7. Venn diagram of differentially expressed metabolites.

https://doi.org/10.3390/microorganisms14030643
